# Supplementary material for: The interactome of intact mitochondria by cross-linking mass spectrometry provides evidence for coexisting respiratory supercomplexes
Source: Mol Cell Proteomics. 2017 Dec 8;17(2):216–32. doi: 10.1074/mcp.RA117.000470 (PMC5795388; doi:10.1074/mcp.RA117.000470)
Supplement: Supplemental Data [file supp_RA117.000470_133922_0_supp_23978_fzffwf.zip › spectra_annotation/mito_spectra_annotation/347-1-3-1-12-1.pdf]

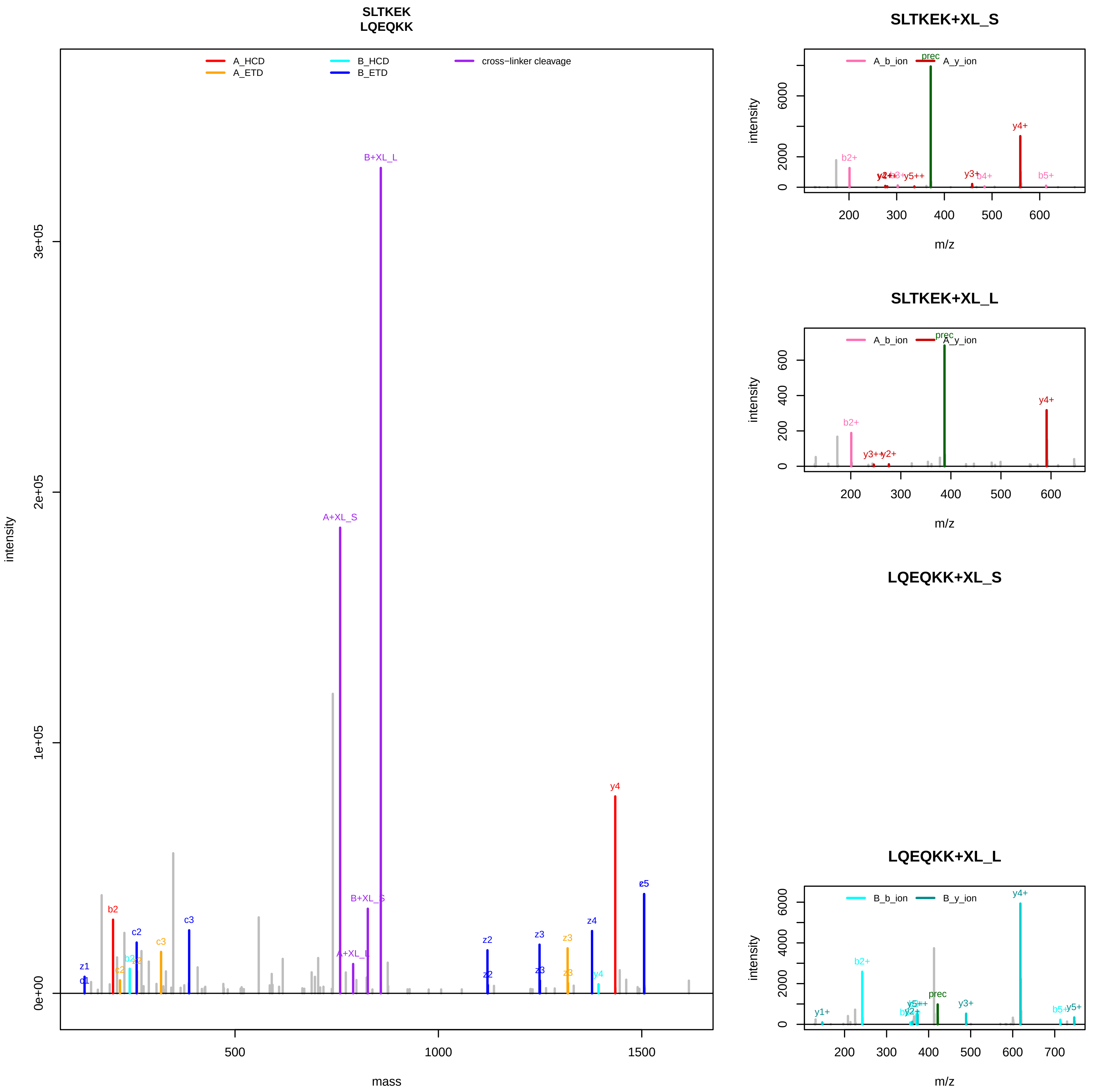

SLTKEK
LQEQKK
A_HCD
A_ETD
B_HCD
B_ETD
cross−linker cleavage
B+XL_L
A+XL_S
B+XL_S
A+XL_L
y4
c5
z5
z4
z3
z2
c3
c2
b2
z1
c1
intensity
mass
3e+05
2e+05
1e+05
0e+00
500
1000
1500
SLTKEK+XL_S
SLTKEK+XL_L
LQEQKK+XL_S
LQEQKK+XL_L
A_b_ion
A_y_ion
B_b_ion
B_y_ion
prec
y4+
y3+
b2+
b4+
b5+
y5++
y3++
y2+
y1+
b3+
b5+
y5+
m/z
6000
4000
2000
0
600
400
200
100
200
300
400
500
600
700
